# Empirical Evidence of Arsenite Oxidase Gene as an Indicator Accounting for Arsenic Phytoextraction by *Pteris vittata*

**DOI:** 10.3390/ijerph19031796

**Published:** 2022-02-04

**Authors:** Ning Han, Chongyang Yang, Shunya Shimomura, Chihiro Inoue, Mei-Fang Chien

**Affiliations:** 1 Graduate School of Environment Studies (GSES), Tohoku University, Sendai 980-0845, Japan; han.ning.p2@dc.tohoku.ac.jp (N.H.); yangchongyang@g.ecc.u-tokyo.ac.jp (C.Y.); chihiro.inoue.b1@tohoku.ac.jp (C.I.); 2 Agro-Biotechnology Research Center, Graduate School of Agricultural and Life Sciences, The University of Tokyo, Tokyo 113-8657, Japan; 3 School of Engineering, Tohoku University, Sendai 980-0845, Japan; nonbiriunderson@gmail.com; 4 Graduate School of Frontier Sciences, The University of Tokyo, Kashiwa 277-8561, Japan

**Keywords:** arsenic, microbe-assisted phytoextraction, *Pteris vittata*, *aioA*-like genes, field trial

## Abstract

Arsenic (As) is a toxic semi-metallic element that is ubiquitous in the environment and poses serious human health risks. Phytoextraction by *Pteris vittata* is considered a low-cost and environmentally friendly approach to treat As-contaminated soil. *P. vittata* mainly absorbs arsenate thus the bioavailability of As to *P. vittata* depends on the chemical form of As. Microbial redox of As contributes to the biogeochemical cycling of As, and rhizobacterium-assisted phytoextraction by *P. vittata* was proposed. In this study, this microbe-assisted phytoextraction was applied to two fields, and the effectiveness of phytoextraction was evaluated. The results revealed that *P. vittata* was able to grow in temperate and subarctic climate zones. The biomass was influenced by the weather, and the As concentration in plants was dependent on the As content in the soil. The ratio of arsenite oxidase genes (*aioA*-like genes) to 16S rRNA genes was employed to evaluate the effect of As phytoextraction, and the results exhibited that the ratio was related to the As concentration in *P. vittata*. Our results showed that arsenite oxidation in the rhizosphere might not be achieved by single-strain inoculation, while this study provided empirical evidence that the rhizospheric *aioA*-like genes could be an indicator for evaluating the effectiveness of As phytoextraction.

## 1. Introduction

Arsenic (As) is a hazardous semi-metallic element widely distributed in the soil environment due to natural sources such as weathering and microbial activity [1]. As is released into the environment anthropogenically as a consequence of using As-based pesticides or mining and smelting activities, which is thereby causing serious As pollution in soil and groundwater [2]. Inorganic As is the main form of As in the soil and is categorized as a group 1 human carcinogen by the International Agency for Research on Cancer (IARC) [3]. Acute As poisoning can even cause respiratory failure, while chronic exposure to As can increase the cancer incidence rate and various cardiovascular diseases [4,5]. Human exposure to As is mainly due to the food chain, including ingestion of As-contaminated drinking water and food [6]. Among all food categories, rice consumption contributes the most to the dietary intake of As [7]. Thus, the risk of As is a major concern in Asian countries that rely on rice as a staple food.

The standard of As is less than 15 mg/kg for farmland and less than 0.01 mg/L for underground water in Japan [8]. The environmental quality standards are similar worldwide. However, a significant proportion of areas around the world contain excessive soil As content and, thus, are recognized as As-contaminated regions. Therefore, remediation of As-contaminated soil is now a matter of urgency. Although the current approaches, such as soil replacement and soil isolation, are considered feasible and efficient [9], the risk of secondary pollution and high energy requirements by those approaches cannot be ignored. A cost-efficient and environmentally friendly strategy is preferred to overcome these disadvantages of current remediation approaches. Phytoremediation is such a strategy which is low-cost and appropriate for widespread pollution [10]. Phytoextraction is a common subprocess of phytoremediation, in which plants remove the contaminant from the soil by uptake and accumulating it in their tissues [11]. The plants that can accumulate large quantities of specific elements and facilitate phytoextraction are called hyperaccumulators [12]. In the case of As, *Pteris vittata* has been reported as the first hyperaccumulator. *P. vittata* is a fern species belonging to the Pteridaceae family and is commonly known as Chinese ladder brake. It can accumulate up to 22,630 mg/kg of As and opens up new possibilities for As phytoremediation [13]. However, several reports indicated that it might take decades to use *P. vittata* to achieve the remediation goals [14,15]. Thus, appropriate measures and strategies should be considered to speed up the process to improve the efficiency of As phytoextraction.

It is known that *P. vittata* prefers absorbing arsenate, As(V), than arsenite, As(III) [16], while As mobilization and transformation in the soil environment mainly occur through microorganisms [17]. Some As-oxidizing rhizobacteria that contain the arsenite oxidase gene, *aioA*, were isolated and reported to be helpful in As hyperaccumulation by *P. vittata* [18,19]. Among them, the *Pseudomonas vancouverensis* strain m318, isolated from the rhizosphere of another As hyperaccumulator *Pteris multifida,* was reported for its multifunctional assistance in As phytoextraction such as its IAA-producing, siderophore-producing, and As(III)-oxidizing abilities. The enhancement of As uptake by *P. vittata* inoculated with strain m318 was verified through a long-term field trial, which showed its potential as an effective agent for microbe-assisted phytoextraction [20]. However, our previous study was mainly focused on the efficacy of strain m318 in a normal fallow field with moderate As content. The performance of strain m318 in assisting phytoextraction under high As-contaminated soil is still unknown. In addition, the effectiveness of microbe-assisted phytoextraction may readily be affected by other environmental factors, including soil composition and climate conditions. Hence, a common indicator independent of environmental factors that can comprehensively evaluate this technology is needed; no such indicator has been available until now. In this study, strain m318 was applied to *P. vittata* in two fields with different As content to test its effectiveness in promoting As uptake. The progress of As phytoextraction was investigated, and an indicator accounting for microbe-assisted phytoextraction was determined.

## 2. Materials and Methods

### 2.1. Site Location and Field Design

**Figure 1:**
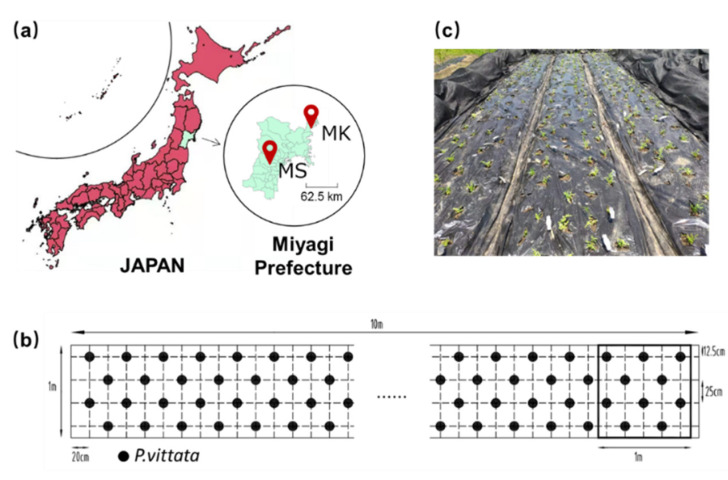
(**a**) Location of two sites in this study (**b**) planting design of *P. vittata* (**c**) photo after transplanted.

The field trials in this study were conducted during 2019–2020. In each year, the plants were planted in June and harvested in October. Two sites in the northeast region of Japan were selected for the co-cultivation experiment. The locations of the experimental fields and planting design are shown in Figure 1.

The first site, MS, is a fallow field located in Sendai city, Miyagi, Japan (38°17′23.4″ N, 140°46′52.2″ E), and the total As content was about 8.6 mg/kg. The MS site is in a temperate zone with an average annual temperature of 13.5 °C and mean annual precipitation of 1249.6 mm. This is the same field as our previous studies [18,20]. Another site, MK, is located in Kesennuma, Miyagi, Japan (38°48′20.6″ N 141°32′04.3″ E), which is contaminated with a high As content of about 60 mg/kg. The MK site is in a subarctic zone with an average annual temperature of 11.4 °C and mean annual precipitation of 1348.6 mm. *P. vittata* is native in tropical and subtropical regions and not cold-tolerant. Therefore, the cultivation period was decided from June to October with the average temperature above 15 °C. The major elemental composition of the soils is shown in Supplementary Material Table S1.

### 2.2. Plant and Microbe Materials and Inoculation Experiment

The plug seedlings of *P. vittata* with 6–9 fronds used in this study were provided by Fujita Co., Ltd. (Tokyo, Japan). For the co-cultivation experiment, *Pseudomonas vancouverensis* strain m318 was inoculated to the rhizosphere of *P. vittata*. The detailed characteristics of this strain were described in our previous study [18]. The m318 strain stored in a −80 °C deep freezer was first pre-cultured in 5 mL of 0.2× Tryptic Soy Broth (TSB) medium with agitation at 120 rpm and 30 °C overnight. Then, 1 mL bacterial solution was transferred into 500 mL of 0.2× TSB medium and cultured at 30 °C with 120 rpm agitation until OD_600 nm_ reached 1.5.

For inoculation treatments (PvI), 5 mL of bacterial culture was added to the rhizosphere of each plant, and for control groups (PvN), 5 mL of 0.2× TSB medium was added instead of bacterial culture. These plants were incubated overnight at room temperature before being transplanted into the field.

### 2.3. Plant Sampling and Analysis

During the cultivation period, plant sampling was conducted in June, August, September, and October. Plants were first shaken to remove the bulk soil, washed with running water, and then rinsed 2–3 times with Milli-Q water. Each plant was divided into shoots and roots and then dried in an oven at 60 °C for 7 days. The dry weight of shoots and roots was measured and recorded separately as the plant biomass. Afterward, the plants were ground, and 0.03 g of well-ground plant materials was added into a 20 mL test tube containing 3 mL of 60% HNO_3_ (Wako, Japan). The tubes were incubated at 130 °C in an aluminum block bath (Scinics Corporation, Tokyo, Japan) for about 2 h until there was no precipitation and no brown smoke came out. The ingredients were transferred to a volumetric flask and diluted to 10 mL with Milli-Q water as the digested solution. For the test solution, 1 mL of digested solution was added with 0.5 mL of 60% HNO_3_, and 10 μL of internal standard (10 mg/mL Indium), followed by making it up to 10 mL with Milli-Q water. Finally, the test solution was passed through a 0.45 μm filter, and the As concentration of the test solution was measured by inductively coupled plasma mass spectrometry (ICP-MS) (NexION 2000, PerkinElmer, Co., Ltd., Waltham, MA, USA).

### 2.4. Soil Sampling and As Content Analysis

Soil samples were collected by using a 30-cm core sampler (FUJIWARA SCIENTIFIC CO., LTD., Tokyo, Japan) at the initial stage (June) and the harvesting stage (October). Soil was sampled from five randomly chosen spots across each of the experimental sites. The soil from the upper 15 cm of the core was sieved through a 2 mm sieve (SIEVE FACTORY IIDA Co., Ltd., Osaka, Japan) to remove stones and other organic debris, then mixed and dried in an oven at 60 °C for 3 days. After drying, the soil was crushed into powder by a Hi-speed Vibrating Sample Mill (Cosmic Mechanical Technology. Co., Ltd., Saitama, Japan). The total As content in the soil was measured with X-ray fluorescence (XRF) according to a previous study [21]. Briefly, 3.0 g of crushed soil powder was filled into a PVC ring and pressurized with a hydraulic press (Manual Hydraulic Press—15 and 25 Ton, Specac, Orpington, UK) to produce a disc-shaped pellet. The pressurization conditions were 100 kN for 1 min, 150 kN for 2 min, and then 200 kN for 5 min. The As content in the pellets was then analyzed with XRF (PANalytical Epsilon5, Almelo, The Netherlands)

### 2.5. Relative Abundance of Arsenite Oxidase Genes (aioA-like Genes) to 16S rRNA Genes Analysis

Rhizospheric soil, defined as soil tightly attached to root surface, was collected for rhizospheric microbial activity analysis. Total soil microbial DNA was extracted from 0.5 g of rhizospheric soil using DNeasy Power Soil Kit (Qiagen, Hilden, Germany). DNA quality and quantity was checked with 1% agarose gel electrophoresis and a Nano Photometer (C40, Implen GmbH, München, Germany). The abundance of total bacteria and bacteria-derivative *aioA*-like genes was analyzed by quantitative real-time PCR (qPCR) targeted to 16S rRNA genes and *aioA*-like genes using a CFX Connect^TM^ Thermocycler (BioRad, Hercules, CA, USA) following the method described in the previous study [20]. A PCR4-TOPO (Thermo Fisher Scientific, Waltham, MA, USA) derivative plasmid carrying V3-V4 region of 16S rRNA genes and a T-Vector pMD19 (Takara Bio Inc., Kusatsu, Japan) derivative plasmid carrying 540 bp of the *aioA*-like gene were used as standards, respectively.

The primers Eu341F (5′-CCTACGGGAGGCAGCAG-3′) and Eu518R (5′-GTATTACCGCGGCTGCTGG-3′) were used to amplify the V3-V4 region of bacterial 16S rRNA genes with the qPCR program as follows: 95 °C for 30 s, then 40 cycles of 95 °C for 5 s, 55 °C for 30 s, and 72 °C for 30 s. The primers aroA95F (5′-TGYCABTWCTGCAIYGYIGG) and aroA599R (5′-TCDGARTTGTASGCIGGICKRTT) were used to amplify the bacterial *aioA*-like genes with the qPCR program as follows: 95 °C for 30 s, then 40 cycles of 95 °C for 45 s, 50 °C for 45 s, and 72 °C for 30 s.

### 2.6. Statistical Analysis

For plant samples, three and four replications were used in 2019 and 2020, respectively. Two technical replicates were performed for qPCR analysis. The data were presented as the mean values of all the replicates with standard error. The one-way analysis of variance (One-way ANOVA) was performed using Origin Pro 2021 (Origin Lab Corp., Northampton, MA, USA), while a statistically significant difference was considered at *p* < 0.05.

## 3. Results and Discussions

### 3.1. Soil As Content in Two Sites

The As content of bulk soil in two sites at the initial and harvest stage of cultivation was evaluated using XRF (Table 1). The As content in the MS site was found to be less than 10 mg/kg, while about 60–70 mg/kg was found in site MK. Tsuchiya et al. reported that the soil in Japan contains an average of 7 mg/kg of naturally occurring As [22]. The high As content in the MK site might be attributed to the presence of an abandoned gold mine nearby. Since the environmental quality standard for soil As pollution is less than 15 mg/kg [23], the MK could be regarded as a high As-contaminated site. Higher naturally occurring As content was reported around MK [24]. Moreover, the abandoned gold mines near the MK site were severely damaged during the 2011 Tōhoku earthquake and tsunami, resulting in the flowing out of a large amount of As-containing sediments that perhaps eventually caused the MK site to become a high As area [22].

### 3.2. Growth of P. vittata in Two Sites

**Figure 2:**
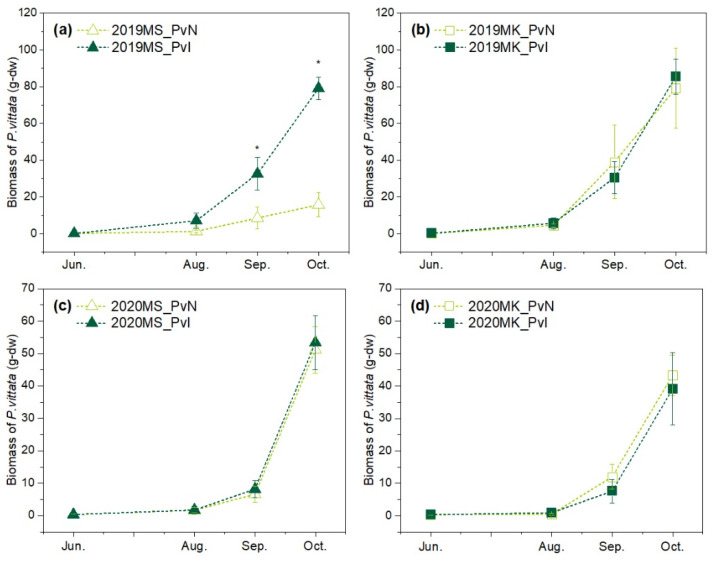
Biomass of *P. vittata* in (**a**) site MS in 2019 (**b**) site MK in 2019 (**c**) site MS in 2020, and (**d**) site MK in 2020. * indicate significant differences (*p* < 0.05) according to ANOVA by Tukey HSD test.

The dry weight of shoots and roots of *P. vittata* across two years is presented in Figure 2. The MS and MK sites shared the same growth trend that no obvious increase for the first three months as an adaptation period, whereas the biomass increased swiftly from September to October. This growth trend was found to be consistent with our previous study [20], while an exception was observed for PvN in MS. Different from the others, the plants under PvN treatment in MS in 2019 did not grow up, as the biomass in October was observed as only 15.77 g-dw. This phenomenon appeared not only for the sampled plants but also for the whole ridge of PvN treatment. Besides, the difference in biomass between the two treatments was confirmed in August, which was still during the early stage of cultivation (Figure 2a). Both above events implied that some environmental stress existed in the early stage of cultivation and resulted in bad colonization of *P. vittata* seedings to MS in 2019. On the other hand, PvI grew well in MS in 2019, implying that strain m318 might have helped *P. vittata* to resist environmental stress.

As for the growth of *P. vittata*, the initial biomass was 0.17 g-dw, and the biomass at the harvest stage was 79.17 g-dw in MS and 85.37 g-dw in MK in 2019 (PvI). In 2020, the initial biomass was 0.22 g-dw, and the biomass of PvI at the harvesting stage was 55.38 g-dw in MS and 39.15 g-dw in MK. These results showed that the biomass was mainly varied with year rather than fields and treatment conditions. *P. vittata* is a fern with low nutritional requirements [25], and thus the climatic conditions could be a major factor affecting its biomass. MS was in a temperate climate zone, and the average temperature in October was recorded as 15.9 °C, which was higher than the recorded average temperature (13.9 °C) in October at MK located in a subarctic climate zone. Since *P. vittata* is native to tropical areas and grows better in a warm environment, the low biomass in MK might have resulted from the colder temperature. Altogether, the current results indicated that *P. vittata* is capable of growing in temperate to subarctic climate zones and can be applied to As phytoextraction.

In addition, the biomass of each *P. vittata* in both MS and MK did not show a significant difference between PvI and PvN in 2020. The biomass yield per square meter was measured and is shown in Table 2. Although the difference in biomass of each *P. vittata* was not observed, the biomass yield in one square meter was increased by inoculation with m318. Considering all the treatment was in the same planting density, it showed that inoculation with strain m318 could greatly improve the survival rate of *P. vittata*.

In our previous study, strain m318 was confirmed to produce IAA at a high level of 12.45 mg·L^−1^·d^−1^ [18]. IAA is a kind of auxin that can promote plant growth by enhancing cell elongation and differentiation [26]. Furthermore, IAA was proven to help plants resist environmental stress such as drought and salinity [27,28]. The results in 2019 indicated the potential ability of strain m318 to promote the survival of *P. vittata* under environmental stress. Another study reported that some plant growth-promoting rhizobacteria (PGPR) capable of producing IAA contributed to wheat yield per hectare, but had no significant effect on each plant [29]. Since strain m318 is an IAA-producing bacterium, it may enhance the survival rate of *P. vittata* by improving its resistance to environmental stress and subsequently increasing the total biomass yield. Further studies to clarify the interactions among IAA-producing rhizobacteria, *P. vittata* and soil during plant growth will be helpful in designing an efficient As phytoextraction strategy.

### 3.3. As Accumulation by P. vittata in Two Sites

**Figure 3:**
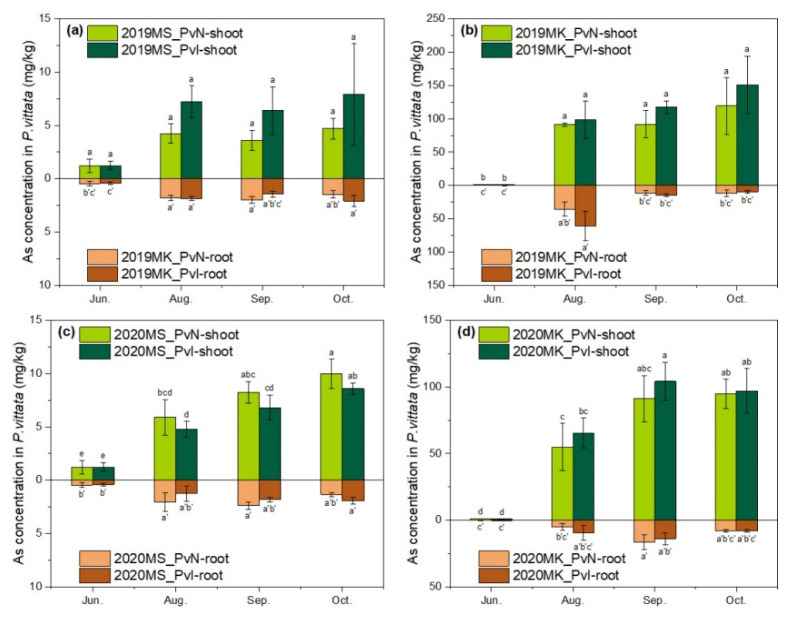
Arsenic concentration in shoot and root of *P. vittata* in (**a**) site MS in 2019 (**b**) site MK in 2019 (**c**) site MS in 2020 (**d**) site MK in 2020. Data with different letters indicated significant differences (*p* < 0.05) according to ANOVA by Tukey HSD test.

**Figure 4:**
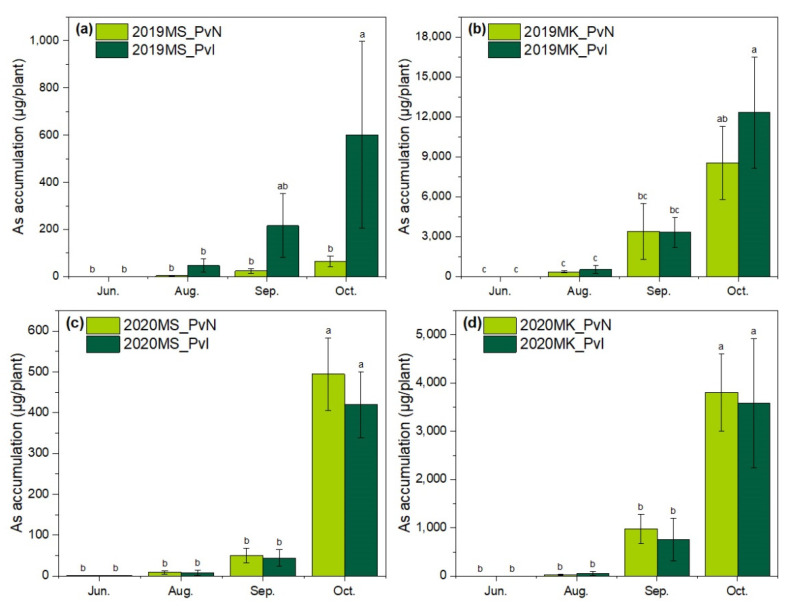
Arsenic accumulation in a *P. vittata* plant in (**a**) site MS in 2019 (**b**) site MK in 2019 (**c**) site MS in 2020, and (**d**) site MK in 2020. Data with different letters indicated significant differences (*p* < 0.05) according to ANOVA by Tukey HSD test.

As concentrations in the shoots and roots are presented in Figure 3. The total As accumulation in each plant was calculated according to the As concentration and dry weight and is shown in Figure 4.

Arsenic concentration in the shoots increased at an early stage of the cultivation period and even further increased with cultivation time, while the As concentration in the roots temporarily increased and became steady (Figure 3). These results supported that As was absorbed by the roots but transferred and accumulated to the shoots, which is consistent with the study reported by Singh and Ma [30].

In 2019, As concentration in the shoots of *P. vittata* with strain m318 inoculation was higher than that without inoculation in both MS and MK exhibiting no statistically significant difference between the sites. This trend was consistent with our previous studies [20] and was also confirmed in MK in 2020. However, the As concentration in the shoot with inoculation was lower than that without inoculation in MS in 2020 (Figure 3c). This phenomenon might be accounted for by the lack of bioavailable As that is related to arsenic redox bacteria in the rhizosphere.

The initial As amount in each *P. vittata* seedling was 0.21 μg. At the harvesting stage (October), the highest As accumulation per plant in site MK was 9371.04 μg (2019-PvN), which was 8.23 times higher than that in site MS (1138.28 μg, 2019-PvI). Since As content in MK soil was approximately seven times higher than MS, these results showed that the As concentration in *P. vittata* was mainly dependent on the As content in the soil. Besides, the similar yield between MS and MK suggested that the high As content in MK had no effect on *P. vittata* growth.

The As removal amount in one square meter was also measured (Table 2). The results showed that PvI treatment removed more As in 2020 in both MS and MK. In 2020, the As removal amount of PvI was 1.2 times higher than that of PvN in site MS, and 1.6 times higher in site MK. Despite the higher As accumulation in each plant of PvN in site MS, the As removal of PvI was much more per unit area due to more plants surviving during the whole cultivation period. This suggested that increasing the survival rate of *P. vittata* could improve the overall remediation efficiency in actual As-contaminated soil.

### 3.4. Arsenite Oxidase Genes in the Rhizosphere of P. vittata

**Figure 5:**
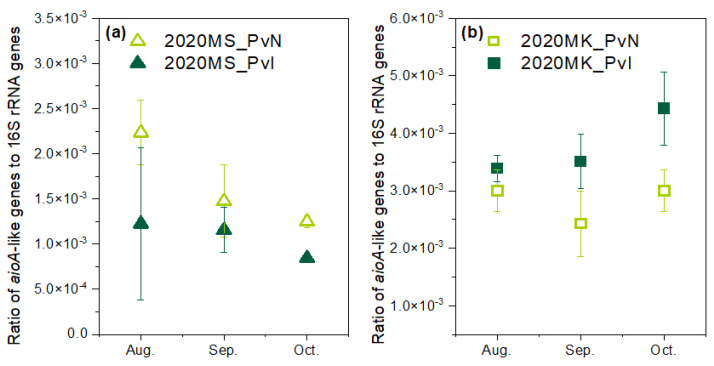
Copy ratio of *aioA*-like genes to 16S rRNA genes in the rhizosphere of *P. vittata* (**a**) 2020 in site MS and (**b**) 2020 in site MK.

To understand the relevance between As uptake and the alteration of rhizospheric microbial communities, the relative abundance of arsenite oxidase genes (*aioA*-like genes) to 16S rRNA genes in the rhizosphere of *P. vittata* in 2020 was investigated. The results observed different tendencies between these two sites (Figure 5). In MK, the relative abundance of *aioA*-like genes under inoculated treatment was higher than that under non-inoculated treatment and kept increasing during the whole cultivation period. It could be speculated that strain m318 was widely colonized in the rhizosphere, or the inoculation of strain m318 activated other microorganisms harboring *aioA*-like genes and impacted the indigenous soil microbial community, forming a synergistic effect that contributed to As uptake by *P. vittata*.

Contrary to the results observed in site MK, the ratio of the *aioA*-like gene under both inoculation and non-inoculation treatments was decreased in MS, and the ratio under inoculation treatment was lower than that under non-inoculation treatment. This trend was completely opposite to the results of our previous 3-year field trial in MS during 2016–2018, in which the ratio of *aioA*-like genes under inoculated treatment was higher than that observed under non-inoculated treatment [20]. It is worth mentioning that the As concentration in the shoots of *P. vittata* was also lower under inoculated treatment than that under non-inoculated treatment in site MS, which was also found to be against our previous study. These phenomena indicated a correlation between the As concentration in shoots and the relative abundance of *aioA*-like genes in the rhizosphere.

Since *P. vittata* absorbs arsenic as As(V), *aioA*-like genes play an important role in phytoextraction by *P. vittata* [31,32]. In the present study, the decrease of *aioA*-like genes in the rhizosphere indicated a possible prohibition of As(III) oxidation resulting in the decrease of As(V) in the rhizosphere and finally causing the reduction of As uptake by *P. vittata*. Our field trial of *P. vittata* in site MS from 2016 to 2020 found that the As concentration in *P. vittata* decreased from 16 to 8.59 mg/kg-dw along with the decrease of the abundance of *aioA*-like genes in the rhizosphere over three years [20]. Hence, the decrease in soil fertility after years of cultivation might be one of the reasons for the decrease of As concentration in *P. vittata*.

In our previous study in site MS during 2016–2018 [20], the inoculation of strain m318 increased the abundance of *aioA*-like genes in the rhizosphere. However, such an increase was found to fluctuate in 2020, as indicated by the ratio of *aioA*-like genes that decreased during the cultivation period (Figure 5a). Generally, the inoculation of exogenous microorganisms to soil would stimulate and lead to perturbation in the native soil microbial communities [33]. However, native microbial communities have resistance to exogenous microorganisms, which might be buffering mechanisms of the ecosystem, especially in the case of direct inoculation of bacterial suspension without a proper carrier [34]. The reasons accounting for the reduction of *aioA*-like genes in the rhizosphere could be presumed to be the result of native soil microbial communities resistance to strain m318. Thus, it was difficult for strain m318 to become dominant in the rhizosphere. The long-term impacts of inoculation on indigenous soil communities are worth investigating in the future.

The abundance of *aioA*-like genes in the rhizosphere seems to be a potential indicator for evaluating the effectiveness of microbe-assisted As phytoextraction by *P. vittata*. It is necessary to determine an approach to increase the relative abundance of the *aioA*-like genes in the rhizosphere. Although inoculating the exogenous microorganisms harboring *aioA*-like genes was considered to be a good choice, our study concluded that the effectiveness of single strain inoculation could be variable. It could be a huge challenge to maintain the long-term effects. Thus, understanding how *P. vittata* assembles its rhizospheric microorganisms when planted in different soil environments could be a key breakthrough. In addition, there are also ecological relationships, including mutualism or competition among soil microorganisms [35]. Some studies indicated that the effect of inoculation with a mixture of multiple microorganisms provided better results than a single strain [36,37]. Therefore, constructing a consortium with strain m318 as the core strain might be a practical approach to promote microbe-assisted As phytoextraction by *P. vittata*.

## 4. Conclusions

In this study, the potentiality of microbe-assisted phytoextraction in different soil conditions on the field scale was evaluated. The growth and As hyperaccumulation of *P. vittata* showed that *P. vittata* is applicable in both temperate/subarctic zones and high/low As soil. Besides, the results showed that strain m318 improved the survival rate of *P. vittata*. Inoculation of strain m318 supported the increase of *aioA*-like genes in the rhizosphere, which contributed to As phytoextraction by *P. vittata*. Though other factors were probably involved in the change of *aioA*-like genes in the rhizosphere, the present study provides empirical evidence that the relative abundance of *aioA*-like genes in the rhizosphere correlated to the As concentration in *P. vittata*. The findings of this study shed light on *aioA*-like genes as an indicator in evaluating the efficiency of microbial-assisted As phytoextraction by *P. vittata*.

## Figures and Tables

**Table 1:** The total As content in soil of experimental sites at the initial and harvesting stages.

	2019						2020	
	MS		MK		MS		MK	
Initial As content (mg/kg)	8.66	±0.67	63.45	±0.69	8.53	±0.72	62.50	±11.93
Harvest As content (mg/kg)	6.50	±1.36	72.54	±8.34	9.64	±1.15	71.83	±13.89

**Table 2:** The parameters regarding As removal per square meter.

	2019				2020			
	MS		MK		MS		MK	
	PvN	PvI	PvN	PvI	PvN	PvI	PvN	PvI
Biomass (kg/m^2^)	NA	0.50	0.52	0.82	0.36	0.48	0.28	0.44
Average As concentration (mg/kg)	4.69	7.91	119.13	150.8	9.56	8.59	94.77	96.89
Removal amount of As (mg/m^2^)	NA	3.96	61.95	123.66	3.44	4.12	26.54	42.63

NA: not available. The biomass of PvN in site MS in 2019 could not be measured due to the growth situation.

## Data Availability

The data presented in this study are available on request from the corresponding author upon reasonable request.

## Supplementary Materials

The following supporting information can be downloaded at: https://www.mdpi.com/article/10.3390/ijerph19031796/s1, Table S1: Soil chemical composition of two sites measured by X-ray fluorescence (XRF).
