# Supplementary material for: Empirical Evidence of Arsenite Oxidase Gene as an Indicator Accounting for Arsenic Phytoextraction by Pteris vittata
Source: Int J Environ Res Public Health. 2022 Feb 4;19(3):1796. doi: 10.3390/ijerph19031796 (PMC8835403; doi:10.3390/ijerph19031796)
Supplement: Supplementary file 1 [file ijerph-19-01796-s001.zip › ijerph-1547915-supplementary.pdf]

Table S1 Soil chemical composition of two sites measured by X-ray fluorescence

Chemical compound	Composition (%)	
	MK	MS
Na_2O	1.10	2.30
MgO	2.09	0.61
Al_2O_3	19.35	15.96
SiO_2	59.07	73.96
P_2O_5	0.22	0.46
K_2O	1.70	0.74
CaO	1.18	2.90
TiO_2	0.89	0.67
MnO	0.07	0.11
Fe_2O_3	7.15	3.99

(XRF)
